# Alteration of Hepatic Gene Expression along with the Inherited Phenotype of Acquired Fatty Liver in Chicken

**DOI:** 10.3390/genes9040199

**Published:** 2018-04-08

**Authors:** Yonghong Zhang, Zhen Liu, Ranran Liu, Jie Wang, Maiqing Zheng, Qinghe Li, Huanxian Cui, Guiping Zhao, Jie Wen

**Affiliations:** 1 Institute of Animal Sciences, Chinese Academy of Agricultural Sciences, Beijing 100193, China; yonghong@jlu.edu.cn (Y.Z.); liuzhen19900708@126.com (Z.L.); liuranran@caas.cn (R.L.); wangjie4007@126.com (J.W.); zhengmaiqing@caas.cn (M.Z.); qli2014@126.com (Q.L.); cuihuanxian78@126.com (H.C.); zhaoguiping@caas.cn (G.Z.); 2 State Key Laboratory of Animal Nutrition, Beijing 100193, China; 3 College of Animal Science, Jilin University, Changchun 130062, China

**Keywords:** chicken, fatty liver, susceptible breed, gene expression, inherited phenotype

## Abstract

Fatty liver is a widespread disease in chickens that causes a decrease in egg production and even death. The characteristics of the inherited phenotype of acquired fatty liver and the molecular mechanisms underlying it, however, are largely unknown. In the current study, fatty liver was induced in 3 breeds by a high-fat (HF) diet and a methionine choline-deficient (MCD) diet. The results showed that the dwarf Jingxing-Huang (JXH) chicken was more susceptible to fatty liver compared with the layer White Leghorns (WL) and local Beijing-You (BJY) breeds. In addition, it was found that the paternal fatty livers induced by HF diet in JXH chickens were inherited. Compared to birds without fatty liver in the control group, both offsprings and their sires with fatty livers in the paternal group exhibited altered hepatic gene expression profiles, including upregulation of several key genes involved in fatty acid metabolism, lipid metabolism and glucose metabolism (*ACACA*, *FASN*, *SCD*, *ACSL5*, *FADS2*, *FABP1*, *APOA4* and *ME1*). This study uniquely revealed that acquired fatty liver in cocks can be inherited. The hepatic gene expression profiles were altered in chickens with the inherited phenotype of acquired paternal fatty liver and several genes could be candidate biomarkers.

## 1. Introduction

Fatty liver, also known as fatty liver syndrome or fatty liver hemorrhagic syndrome, is a metabolic disease which is likely caused by nutritional, hormonal, environmental and genetic factors [1,2,3,4,5]. Although it is widespread in laying hens [6], fatty liver is often ignored because its symptoms are not as obvious as those of infectious diseases. Compared with infectious diseases, the mortality of fatty liver is lower. Nevertheless, it can cause decreased egg production, fertilization rate and hatching rate, which lead to economic losses in the poultry industry [7].

Fat metabolism in the liver is in a dynamic balance under physiological conditions. Lipid homeostasis is disrupted when disorders of metabolic pathways occur, such as lipid absorption, lipid synthesis, β-oxidation and lipoprotein transport, during which lipid accumulates excessively in the liver, thereby leading to fatty liver [8,9].

The study of fatty liver is difficult because it is often irregular and not easy to diagnose before death. Accordingly, it is important to establish an experimental model. Environmental and genetic factors, and their interactions, play important roles in the phenotypic generation and pathogenesis [10,11,12]. Nutritional factors can induce fatty liver in chickens [13]. High-energy, low-protein diet increases hepatic fatty acid biosynthesis and decreases or blocks lipoprotein synthesis. As a result, the ability to transport lipids, as lipoproteins, from liver cells to blood is inadequate, and thus lipids accumulate in the liver. Nutritional factors, including choline, phospholipids, vitamin B and vitamin E, are also important materials in hepatic lipoprotein synthesis [14,15]. Deficiency of those nutrients decreases lipoprotein synthesis or blocks lipid transport, leading to excessive fat accumulation in the liver. Likewise, genetic factors greatly influence the susceptibility to fatty liver [16,17,18]. Accordingly, fatty liver was induced in 3 breeds, namely dwarf Jingxing-Huang (JXH), layer White Leghorns (WL) and local Beijing-You (BJY), by 2 types of diets (high fat (HF) diet and methionine choline-deficient (MCD) diet) in the current study conducted to screen for the best fatty liver chicken model.

Many acquired traits related to fat metabolism are inherited in humans, mice and pigs [19,20,21,22]. Studies have shown that paternal diabetes in mammals could be transgenerationally inherited [23]. Additionally, Bruce et al., found that inducing female mice by high-fat diet led to the development of nonalcoholic fatty liver disease (NAFLD) in offspring and altered gene expression in liver [24]. Unlike in mammals, lipids and lipoproteins are mainly synthesized in the liver of chickens and male chickens are the homogametic sex (ZZ). Thus, the characteristics of the inherited phenotype of fatty liver in chickens are far from clear. In the present study, the effects of parental fatty liver on the offspring and the associated underlying molecular basis were investigated.

## 2. Materials and Methods

### 2.1. Ethics Approval

All the animal experiments were conducted in accordance with the guidelines for experimental animals established by the Ministry of Science and Technology (Beijing, China). Ethical approval on animal survival was given by the animal ethics committee of the Institute of Animal Sciences (IAS, Kaunas, Lithuania), Chinese Academy of Agricultural Sciences (CAAS, Beijing, China) with the following reference number: IASCAAS-AE-03.

### 2.2. Birds and Treatments

**Figure 1:**
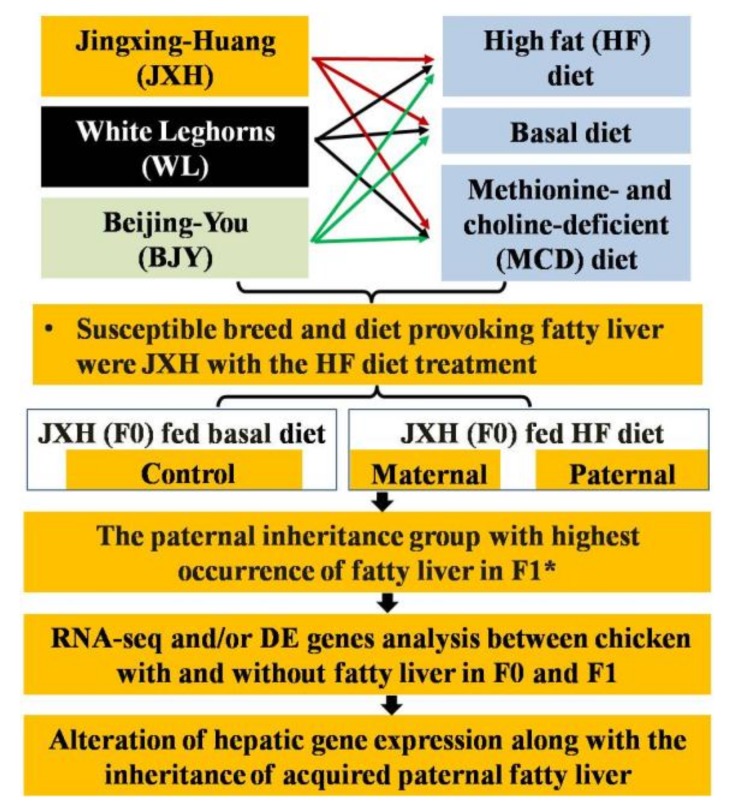
Graphic representation of the experimental procedures and main results. * The F1 chickens of control, maternal and paternal groups were all fed normal diet. DE: differentially expressed.

Three hundred one-day-old chickens from each of the three studied breeds, namely JXH, WL and BJY, were used. The JXH is a cultivated dwarf chicken line, WL is a commercial layer line, and BJY is a native Chinese breed used for meat and egg production. The animals of each group came from a closed population of the same breed. The number of males and females was the same. All birds were reared in stair-step caging using standard conditions of temperature, humidity, light and ventilation. From week 1 to 17, the same diet was fed to all chickens and was formulated to be intermediate among recommendations for the different breeds. The starter ration (week 1 to 8) contained 18.5% crude protein and 2.87 methionen choline (MC)/kg. The ration from week 9 to 17 contained 15.2% crude protein and 2.83 MC/kg. The fatty liver induction experiment was conducted from week 19 to 25 after one week of adaptation feeding. Groups of 120 males and 120 females with comparable body weight were selected from each breed and randomly assigned to three dietary treatments, each with four replicates. For the basal diet group, each replicate had five chickens. For the HF diet and MCD diet, each replicate had 10 chickens. The composition and nutrient levels of the HF, basal and MCD diets given to chickens from week 18 to 25 are listed in Table 1. The basal diet was formulated based on the NRC (1994) and NY/T (33–2004) to meet the nutrient requirements for chickens [25,26]. Feed and water were provided *ad libitum* during the experiment. The time of sample collection for the study with three breeds was at the end of the 25th week. A graphic representation of the experimental procedures is given in Figure 1.

### 2.3. Birds for Analysis of the Fatty Liver Inheritance Pattern

**Figure 2:**
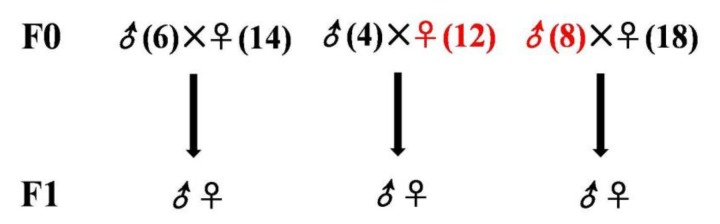
Generation of three cross breeds to examine the inheritance of fatty liver. The control group (**left**) using JXH chickens fed with the basal diet, maternal inheritance group (**middle**) and paternal inheritance group (**right**) using JXH chickens with HF diet-induced fatty liver. Chickens from F0 with fatty liver are shown in red while those without fatty liver are in black. ♂ and ♀ represent the cock and the hen, respectively.

The initial breed and diet comparison indicated that the JXH chickens with the HF diet treatment would be the most appropriate to study the underlying genetics, so no other breeds were used to study the next generation. To investigate the inheritance of fatty liver, this disease was induced in a paternal inheritance group (20♂:60♀) and a maternal inheritance group (9♂:45♀) of JXH chickens with the HF diet, while the JXH chickens of the control group (10♂:50♀) were fed with the basal diet. After reproduction of the F1 generation chickens, the F0 poultry birds were slaughtered and examined to double-check the presence of fatty liver at the end of the 36th week. Ultimately, there were eight males with fatty liver that were bred with 18 females without fatty liver and used for the paternal inheritance study; the four males without fatty liver were bred with 12 females with fatty liver and used for the maternal inheritance study; the six normal males and 14 normal females were used as controls, as shown in Figure 2. The HF diet was introduced from week 18 to 36 for chickens in the paternal and maternal inheritance groups, while the control group received the basal diet of F0 generation. The chickens of the F1 generation were raised with the basal diet without induction of fatty liver (Table 2). The time of sample collection from the F0 and F1 generation of the JXH chickens for inheritance pattern study was at the end of the 36th week.

### 2.4. Sample Collection

Blood samples were drawn from the left-wing vein, and then serum was isolated after incubation for 1 h at 37 °C. Chickens were stunned and killed by approved methods, then a piece of liver was snap-frozen in liquid nitrogen and stored at −80 °C for RNA extraction. Another piece of liver was fixed for 48 h in 4% paraformaldehyde solution (Beijing Solarbio Science and Technology Co., Ltd., Beijing, China) for histological analysis using hematoxylin-eosin (H&E) and oil red O staining. 

### 2.5. Serum Indices and Histology

The triglyceride (TG), total cholesterol (CHOL), low-density lipoprotein (LDL-CHOL) and high-density lipoprotein (HDL-CHOL) concentrations in the serum were measured by North Biological Technology Co., Ltd. (Beijing, China). Fixed liver samples were processed for sectioning and then stained with H&E or oil red O by Beijing Xuebang Science and Technology Co., Ltd. (Beijing, China).

### 2.6. Assessment of Fatty Liver

The degree of fatty liver was assessed by examining the H&E and oil red O histopathology. The extent of fatty degeneration was graded from −33 to 50% (mild), 50 to 75% (moderate) and >75% (severe). The whole-liver phenotype and serum indices were also evaluated.

### 2.7. RNA Extraction and Sequencing

The 6 samples used for RNA-seq analysis were obtained from the F1 generation of JXH chickens (3 birds with fatty liver from the paternal group and 3 without from the control group). The same 6 chickens, along with their sires in F0 and 6 additional F1 half-sibs showing the corresponding phenotypes were used for quantitative real time polymerase chain reaction (qRT-PCR) analysis. Total RNA was extracted from frozen liver using an RNA Isolation Kit (TIANGEN, Beijing, China). After treatment with RNase-Free DNase I (New England Biolabs, Ipswich, MA, USA), total RNA was extracted with phenol-chloroform and precipitated with ethanol. The quality and concentration of the total RNA were determined with a NanoDrop 2000 spectrometer (Thermo Fisher Scientific, Waltham, MA, USA) and its integrity was further verified by agarose gel electrophoresis. mRNA was enriched from the total RNA using oligo-d(T) probes for polyA selection. The enriched mRNA was fragmented into short fragments of around 200 bp in fragmentation buffer. The fragmented mRNA was converted into complementary DNA (cDNA) using random hexamers. The double-stranded cDNA was subsequently subjected to end repair, adapter ligation, and fragment selection. The libraries, generated by PCR amplification, were sequenced by HiSeq 2500 (Illumina, San Diego, CA, USA).

### 2.8. Differential Expression Analysis

The sequenced data was filtered with the Sickle and SeqPrep software to remove the noise and low-quality sequences [27,28]. The clean reads were mapped on the reference genome of *Gallus*_*gallus*_5.0. The clean data generated have been deposited in the GEO and are publicly accessible accession no. GSE111909. The expression abundance of the unigenes was calculated according to fragments per kilobase of exon per million (FPKM) fragments mapped. Differentially expressed genes (DEGs), with a false discovery rate (FDR) < 0.05, were selected as the criteria for significant differences.

### 2.9. Functional Enrichment Analysis of Differentially Expressed Genes

In order to investigate the biological function of the DEGs, gene ontology (GO) enrichment and Kyoto encyclopedia of genes and genomes (KEGG) pathway [29] analyses were conducted using the database for annotation, visualization and integrated discovery (DAVID) v6.7 bioinformatic resources [30], with the statistical significance *p*-value cutoff was set at 0.05.

### 2.10. Quantitative Real-Time PCR 

The PrimeScriptTM RT-PCR Kit (TAKARA, Otsu, Japan) was used for reverse transcription of liver mRNA. Primers were designed based on chicken gene sequences from the National Center for Biotechnology Information (NCBI) database using Primer 5.0 (Table 3) and were synthesized by BGI Sequencing Biological Engineering Technology & Services Co. Ltd. (Beijing, China). The qRT-PCR analysis was performed with the SYBR Premix Ex TaqTM reagent Kit (TAKARA, Kusatsu, Japan) using the Light Cycler 7500 (Roche Applied Science, Penzberg, Germany) to quantify the florescence. The qRT-PCR reaction conditions were as follows: 94 °C for 5 min; followed by 35 cycles of 94 °C for 3 s, at the annealing temperature of 32 s. Each sample was analyzed in triplicate, and the results were quantified by the 2^−ΔΔ*C*T^ method [31].

### 2.11. Statistical Analysis

All statistical analyses were performed with the SPSS 19.0 software (IBM Corp., Armonk, NY, USA). The results are presented as the mean ± standard error of mean. The significance of the observed differences was examined using the *t*-test for two independent samples. The level of significance was set at *p* ≤ 0.05.

## 3. Results

### 3.1. Identification of the Most Susceptible Breed and Diet Causing Fatty Liver

**Figure 3:**
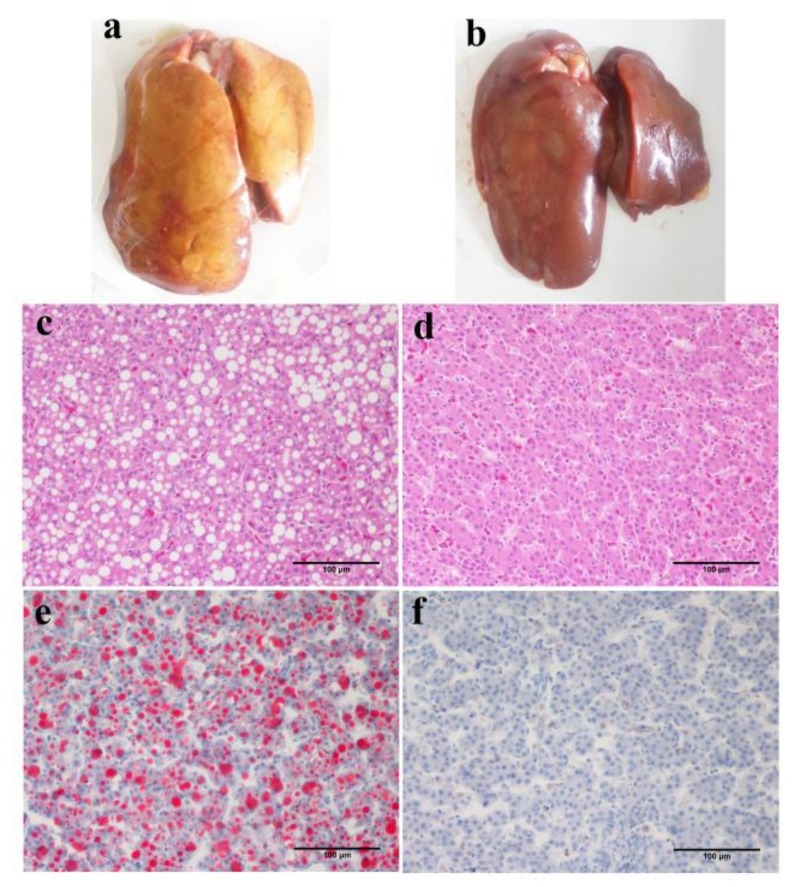
Typical features of fatty liver in terms of gross appearance and sections stained with hematoxylin-eosin(H&E) or oil red O (20×). The fatty liver appears yellow or light yellow and the edge of the liver is blunt and friable (**a**). Enlarged liver cells with abundant fat droplets of different sizes are present in the cytoplasm and liver leaflets lost the normal reticular formation (**c**,**e**). In comparison, the non-fatty liver was dark red with sharp edges, the texture was flexible and there were less fat droplets and clear reticular formations in liver leaflets (**b**,**d**,**f**). (**a**,**c**,**e**) were from one JXH chicken with HF diet; (**b**,**d**,**f**) from one JXH chicken with basal diet. Scale bar: 100 μm.

The occurrence of fatty liver induced by 2 diets in 3 breeds was evaluated, and a summary of the results is shown in Table 4. The typical features of fatty liver are shown in Figure 3. The frequency of occurrence of fatty liver in JXH was higher among the chickens fed with the HF diet (41.9%) and MCD diet (38.7%) than those fed with the basal diet (25%). Meanwhile, in WL chickens, the fatty liver incidence was increased by the HF diet, but not by the MCD diet, while in BJY chickens, fatty liver incidence was moderately increased by the HF and MCD diets (33.33%). Accordingly, the chickens most susceptible to fatty liver were those of the JXH breed, and the diet most effective at causing fatty liver was the HF diet.

### 3.2. Characteristics of Inherited Phenotype of Acquired Fatty LiverInduced by High Fat Diet in Jingxing-Huang Chickens

**Figure 4:**
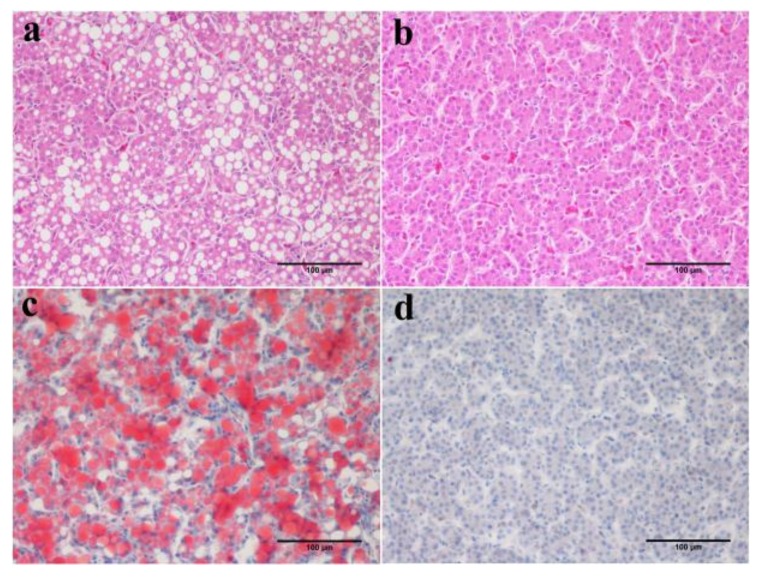
Typical H&E and oil red O stained liver sections (20×) from F1 generation of JXH chicken with or without fatty liver, paternal group (**a**,**c**); control group (**b**,**d**). Scale bar: 100 μm.

**Figure 5:**
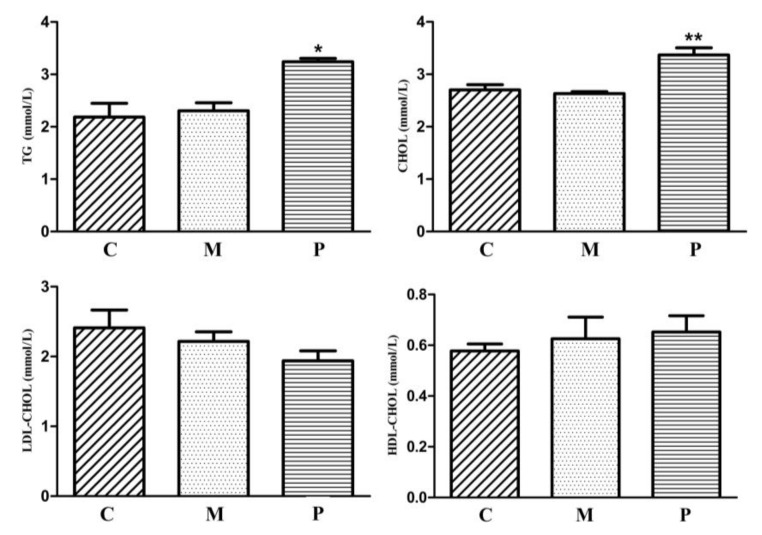
Serum lipid indices of all F1 JXH chickens in the control (C, *n* = 105), maternal (M, *n* = 80) and paternal (P, *n* = 82) groups. TG: triglyceride, CHOL: total cholesterol, LDL-CHOL: low-density lipoprotein, HDL-CHOL: high-density lipoprotein. * indicates *p* < 0.05 and ** indicates *p* < 0.01.

JXH chickens fed with the HF diet were identified as the best model to study fatty liver. The fatty liver paternal inheritance group and maternal inheritance group consisting of JXH chickens were fed with the HF diet to induce fatty liver in the F0 generation, as described earlier, while the control group of JXH chickens were fed with the basal diet in F0. The results showed that the occurrence of fatty liver, without dietary induction, in the F1 generation was highest (41.5%, *n* = 82) in the paternal inheritance group, which were chickens from cocks with fatty liver bred with normal hens (Figure 4, Table 5). In comparison, the frequency of occurrence of fatty liver was considerably lower in chickens that were the progeny of hens with fatty liver bred with normal chickens (20%, *n* = 105), as well as in those from control parents without fatty liver (18.75%, *n* = 80). In addition, consistently, the F1 chickens in the paternal inheritance group had higher serum TG (*p* < 0.05) and CHOL (*p* < 0.01) contents than that of the progeny of the control and maternal inheritance groups. There were no differences in serum LDL-CHOL and HDL-CHOL among the 3 groups (Figure 5).

### 3.3. Differencein Hepatic Gene Expression Profiles between F1 Chickens with and without Fatty Liver

**Figure 6:**
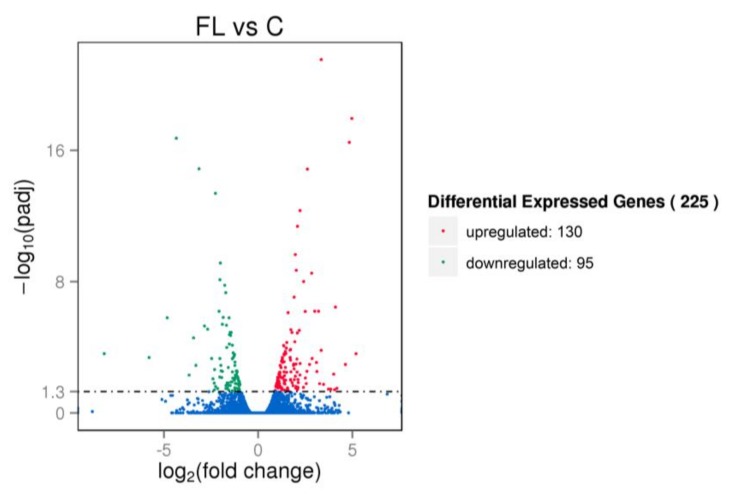
Volcano plot of differentially expressed genes (DEGs). Red, green and blue points represent significantly upregulated, downregulated and non-regulated genes, respectively. The abscissa is the fold change of the genes in different samples, ordinate is the variation difference of the gene expression with statistical significance. FL: paternal inheritance group, C: control group.

The DEGs between F1 chickens with fatty liver from the paternal group and chickens with non-fatty liver from the control group were determined by RNA-seq analysis and the results are presented in Table 6 and Figure 6. From the sequencing of 6 samples, 81,465,412 (fatty liver) and 76,906,656 (non-fatty liver) clean reads were obtained. Ultimately, a total of 225 DEGs were identified, of which 130 were upregulated and 95 were downregulated in birds with fatty liver.

**Figure 7:**
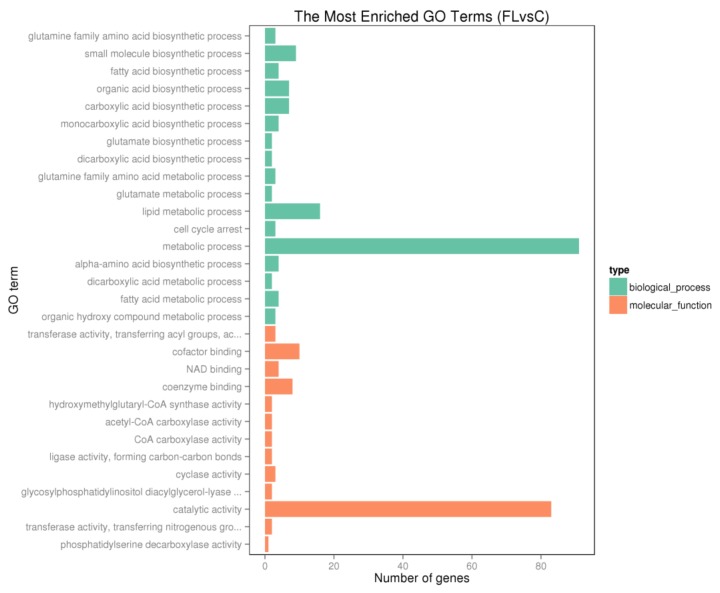
The most enriched gene ontology (GO) terms for the differentially expressed genes.

**Figure 8:**
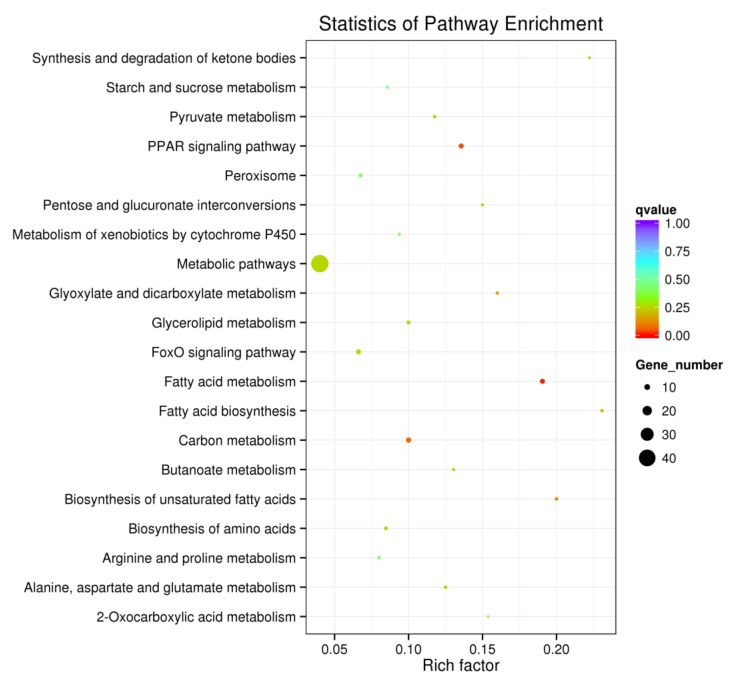
The pathway enrichment analyses for the DEGs.

In order to determine molecular pathways involved in the generation of fatty liver, GO and KEGG pathway analyses were performed with the DEGs. As shown in Figure 7 and Figure 8, there was significant enrichmentof the fatty acid metabolic and biosynthesis pathway (gga01212, gga00061, gga01040, GO:0006631, GO:0006633), lipid metabolic pathway (GO:0008610, GO:0044255, GO:0006629), PPAR signaling pathway (gga03320) and FoxO signaling pathway (gga04068).

### 3.4. Alteration of the Key Gene Expression along with the Inherited Phenotype of Acquired Paternal Fatty Liver

**Figure 9:**
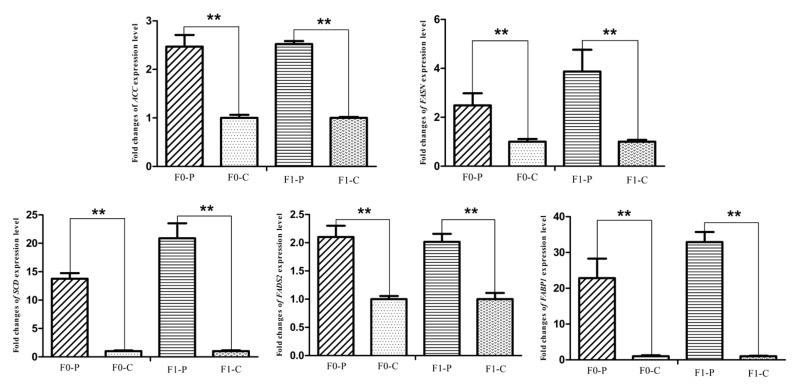
Fold changes of fatty acid metabolism genes (*ACC*, *FASN*, *SCD*, *FADS2*) and lipid metabolism gene (*FABP1*) expression levels between P (paternal inheritance group) and C (control group) in F0 and F1 generations. ** indicates *p* < 0.01.

**Figure 10:**
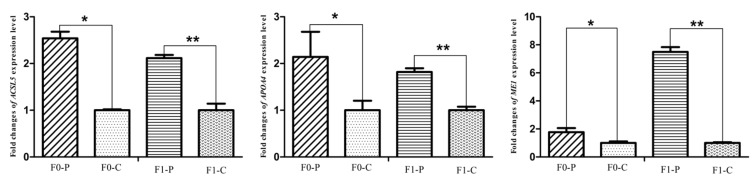
Fold changes of fatty acid metabolism gene (*ACSL5*), lipid metabolism gene (*APOA4*) and glucose metabolism gene (*ME1*) expression levels between P (paternal inheritance group) and C (control group) in the F0 and F1 generations. * indicates *p* < 0.05 and ** indicates *p* < 0.01.

**Figure 11:**
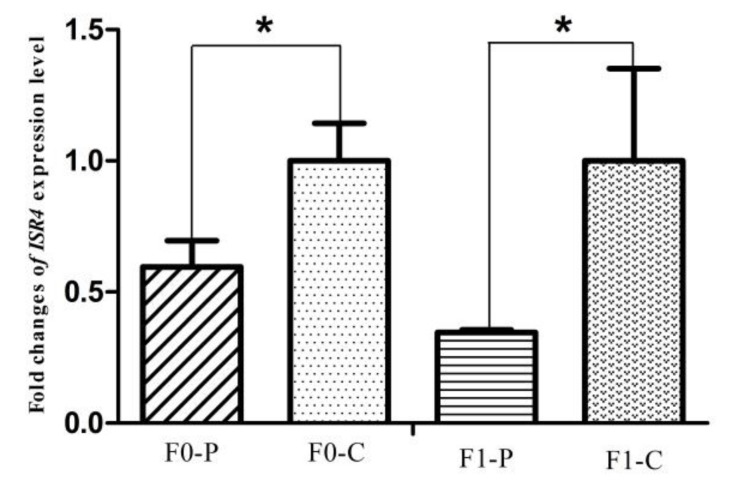
Fold change of glucose metabolism gene, *ISR4*, expression level between P (paternal inheritance group) and C (control group) in the F0 and F1 generations. * indicates *p* < 0.05.

The six samples used for RNA-seq from the F1 generation of JXH chickens, along with their sires in F0 and 6 additional F1 half-sibs showing the corresponding phenotypes were used for qRT-PCR analysis. The relative abundance of the DEG transcripts above related to fatty acid metabolism (*ACACA*, *FASN*, *SCD*, *ACSL5* and *FADS2*), lipid metabolism (*FABP1* and *APOA4*) and glucose metabolism (*ISR4* and *ME1*) were measured by qRT-PCR analysis. The transcripts of *ACACA*, *FASN*, *SCD*, *FADS2* and *FABP1* (Figure 9) in chickens with fatty liver were significantly increased compared with those in chickens without fatty liver. This was true in both generations, the F0 chickens with diet-induced fatty liver and the F1 chickens with fatty liver without dietary induction (*p* < 0.01). The expression levels of *ACSL5*, *APOA4* and *ME1* (Figure 10) in F0 chickens with fatty liver were higher than those in chickens without fatty liver (*p* < 0.05), but the differences were highly significant in the F1 generation (*p* < 0.01). In addition, the expression levels of *ISR4* (Figure 11) in both the F0 and F1 generations were significantly lower in chickens with fatty liver than in chickens without the condition (*p* < 0.05).

## 4. Discussion and Conclusions

To establish an experimental model to study fatty liver in chickens, three poultry breeds and two dietary regimens were evaluated in the current study. With either of the two diets used, the JXH chickens were more prone to developing fatty liver compared with the local BJY breed and egg layer WL breed. A possible reason for this is that the JXH is an improved dwarf line with greater capacity for fat deposition [32].

The present study found that both the HF diet and MCD diet could successfully induce fatty liver formation in the JXH and BJY poultry breeds, but the HF diet was more effective, indicating that both the breed and diet type should be considered when establishing a model of fatty liver. The accumulation of TG in the cytoplasm of hepatocytes, the hallmark of fatty liver, arises from an imbalance between lipid acquisition (fatty acid uptake and de novo lipogenesis) and removal (mitochondrial fatty acid oxidation and export as a component of very low-density lipoprotein (VLDL) particles). The HF diet contained more raw materials for fat synthesis, thus consuming it would lead to more hepatic lipogenesis and excessive lipid accumulation, which would in turn result in fatty liver. Choline is a substrate for lipoprotein synthesis, and lipoproteins play an important role in the transmembrane transport of lipids [14,15]. When choline is insufficient, the transport capacity of lipoprotein is impaired, and TGs accumulate in hepatocytes, probably explaining why the MCD diet induces fatty liver. This is also consistent with the finding reported by Polin and Wolford indicating that various types of diets and sources of energy in excess could induce fatty liver hemorrhagic syndrome (FLHS) [33]. In addition, with basal diet, the spontaneous rates of fatty liver observed in the present study were: 16.67% in WL and BJY; and 18.75% or 25% in dwarf JXH of the F1 or F0 generation, respectively. In previous reports, the spontaneous rates of fatty liver have been highly variable in different populations, ranging between 1% and 30% in hens; and have been influenced by nutrients, environment and genetics factors [34,35].

Previous studies have shown the parental effects of fatty liver on the offspring in mammals [23,36], but the impact of parental fatty liver on the progeny in chickens has not been described. In the present study, the acquired paternal fatty liver induced by the HF diet in JXH chickens was found to have a clear impact on the offspring and the condition appeared to be inherited paternally from the F0 to the F1 generation. In addition, the acquired maternal fatty liver was found to have no significant effect on the offspring in the current study, but this might be related to the limited number of families used.

Using a whole-genome RNA-Seq strategy, 225 DEGs were identified between chickens with fatty liver in paternal group and chickens with non-fatty liver in the control group in the F1 generation. The hepatic DEGs related to fatty acid metabolism (*ACACA*, *FASN*, *SCD*, *ACSL5* and *FADS2*), lipid transport (*FABP1* and *APOA4*) and glucose metabolism (*ISR4* and *ME1*) were validated by qRT-PCR in their sires in F0. Consistent changes in expression were found in both the F0 and F1 generations between chickens with fatty liver and chickens with non-fatty liver, indicating the global change of the gene expression profile along with inheritance of the phenotype. In the F0 chickens, the fatty liver was induced by dietary means, but the F1 chickens were not subjected to any dietary induction. The main results of this study are shown in Figure 1. Previously, consistently, Bruce et al. and Dudley et al. found that the offspring born to mothers fed high-fat diets produced a fatty liver phenotype similar to that in non-alcoholic fatty liver disease and hepatic steatosis; they also found that associated genes were significantly changed between maternal high-fat animals and control offspring [24,36]. Wei et al. found that the paternal prediabetic conditions had an impact on the susceptibility in offspring to obesity and type II diabetes. Expression of genes involved in glucose metabolism and insulin signaling pathways in the pancreatic islets of the offspring of prediabetic fathers were also found to be changed [23].

It is well known that acquired traits can be inherited to the next generation through epigenetic regulations [36,37,38,39]. Our previous study found that the abundance of *ACACA* and microsomal triglyceride transfer protein (*MTTP*) transcripts was inversely correlated with the DNA methylation level between fatty liver and normal individuals [5]. Chen et al., reported that sperm tsRNAs (transfer RNA–derived small RNAs) contributed to inheritance of an acquired metabolic disorder [37]. Accordingly, further study is warranted to clarify the epigenetic changes underlying differences in gene expression associated with the inheritance of acquired fatty liver in chickens.

In conclusion, this study uniquely revealed that acquired fatty liver in cocks can be inherited. The hepatic gene expression profiles were altered along with the inherited phenotype of acquired paternal fatty liver in chicken. Additionally, several genes that could be candidate biomarkers were identified. The results will contribute to lay the foundation for further molecular studies on the pathogenesis of fatty liver.

## Figures and Tables

**Table 1:** Composition and nutrient levels of the diets (air-dry basis) %.

Items	Basal Diet	HF Diet	MCD Diet
Ingredients			
Corn	70.10	62.59	70.10
Soybean meal	22.60	20.11	22.60
*DL*-methionion	0.17	0.17	0
*L*-lysine	0.05	0.05	0.05
CaHPO_4_·2H_2_O	1.50	1.50	1.50
NaCl	0.28	0.28	0.28
Limestone	4.30	4.30	4.47
1% premix ^1^	1.00	1.00	1.00
Sheep fat	0	10	0
Total	100	100	100
Nutrient levels ^2^			
GE ^3^, MJ/kg ^4^	18.29	20.32	18.09
CP ^5^	15.93	14.59	15.43
Lys	0.79	0.74	0.80
Met	0.30	0.32	0.18
Ca ^6^	7.24	5.76	8.79
TP ^7^	0.47	0.40	0.44
Choline, mg/kg	1022	886	613

^1^ The premix provided the following nutrients per kg of diet: Fe (as ferrous sulfate) 100 mg, Cu (as copper sulfate) 8 mg, Zn (as zinc sulfate) 80 mg, Mn (as manganese sulfate) 100 mg, Se (as sodium selenite) 0.3 mg, I (as potassium iodide) 1 mg, vitamin A 12000 IU, vitamin D 32400 IU, vitamin E 27 IU, vitamin K 2 mg, thiamine 2 mg, riboflavin 8.0 mg, calcium pantothenate 12.0 mg, nicotinic acid 32.5 mg, pyridoxine 5 mg, VB12 0.2 mg, biotin 0.2 mg. ^2^ The nutrient levels were calculated according to the composition of the raw materials. ^3^ GE: Gross energy. ^4^ MJ/kg: mega joule/kilogram. ^5^ CP: crude protein. ^6^ Ca: calcium. ^7^ TP: total phosphorus.

**Table 2:** The diets for Jingxing-Huang (JXH) chicken in F0 and F1 generations.

Items	1–17 Week			18–36 Week		
	Control	Maternal	Paternal	Control	Maternal	Paternal
F0	Basal diet			Basal diet	HF diet	HF diet
F1	Basal diet					

**Table 3:** Genes and related primers for quantitative real time PCR (qRT-PCR) analysis.

Gene	Accession Number	Primer Sequence	Annealing Temperature, °C	Product Size (bp)
*ACACA*	NM_205505.1	F:5′-AATGGCAGCTTTGGAGGTGT-3′	60	136
		R:5′-TCTGTTTGGGTGGGAGGTG-3′		
*FASN*	NM_205155.2	F:5′-CTATCGACACAGCCTGCTCCT-3′	62	107
		R:5′-CAGAATGTTGACCCCTCCTACC-3′		
*ISR4*	XM_003641084.3	F:5′-GCAAGAAGGGAGTGGAAGGTA-3′	62	121
		R:5′-GCTGGAAGAAACGCTGATAGG-3′		
*ME1*	NM_204303.1	F:5′-GCCAGCATTACGGTTTAGCAT-3′	58.5	90
		R:5′-CCATAACAGCCAAGGTCTCCA-3′		
*SCD*	NM_204890.1	F:5′-GGCTGACAAAGTGGTGATG-3′	60	137
		R:5′-GGATGGCTGGAATGAAGA-3′		
*ACSL5*	NM_001031237.1	F:5′-TTCTCACCGCTCCCAACAC-3′	60	147
		F:5′-TCTTCTGGCTCCTCCCTCAA-3′		
*FADS2*	NM_001160428.2	F:5′-CTGAGGAAGACAGCAGAGGACAT-3′	60	153
		R:5′-GCAGGCAAGGATTAGAGTTGTG-3′		
*FABP1*	NM_204192.3	F:5′-GGGGAAGAGTGTGAGATGGA-3′	58	120
		R:5′-GTTGAGTTCGGTCACGGATT-3′		
*APOA4*	NM_204938.2	F:5′-TCCTCTTGGTGCTCCTGGCTGTG-3′	61	197
		R:5′-GGCGTATGAGTTTGCGCTCTGC-3′		
β-actin	NM_205518.1	R:5′-GAGAAATTGTGCGTGACATCA-3′	60	152
		R:5′-CCTGAACCTCTCATTGCCA-3′		

*ACACA*: acetyl-CoA carboxylase α; *FASN*: fatty acid synthase; *ISR4*: insulin receptor substrate 4; *ME1*: malic enzyme 1; *SCD*: stearoyl-CoA desaturase; *ACSL5*: Acyl-CoA synthetase long-chain family member 5; *FADS2*: fatty acid desaturase 2; *FABP1*: fatty acid binding protein 1; *APOA4*: apolipoprotein A4.

**Table 4:** Results of the evaluation of the fatty liver incidence in three breeds after induction by two diets.

Diet Type	Breed		
	JXH	WL	BJY
Basal diet	25.00%, 8/32	16.67%, 5/30	16.67%, 5/30
HF diet	41.94%, 13/31	33.33%, 10/30	33.33%, 10/30
MCD diet	38.71%, 12/31	16.67%, 5/30	33.33%, 10/30

JXH: Jingxing-Huang; WL: White Leghorns; BJY: Beijing-You; HF: high-fat; MCD: Methionine choline-deficient.

**Table 5:** The incidence of fatty liver in F1 generation of JXH chickens.

Items	Control	Maternal Inheritance	Paternal Inheritance
No. of chicken observed	80	105	82
Rate for the fatty liver incidence	18.75%	20.00%	41.50%

**Table 6:** The output quality data of the transcriptomic sequencing.

Sample	Raw Reads	Clean Reads	Clean Bases, G	Q30, %
P_R1	28,754,076	27,836,516	4.18	92.40
P_R2	27,305,304	26,376,696	3.96	91.81
P_R3	27,879,606	27,252,200	4.09	93.24
C_R1	28,237,546	27,601,240	4.14	93.23
C_R2	26,136,280	25,521,394	3.83	92.95
C_R3	24,399,658	23,784,022	3.57	92.47

P_R: paternal inheritance group; C_R: control group.

## Supplementary Materials

The DEGs between F1 chickens with fatty liver from the paternal group and chickens with non-fatty liver from the control group (XLSX).
